# Pseudoacacia Honey of Hungarian and Slovak Origin: Selected Quality Parameters and Antioxidant Capacity

**DOI:** 10.3390/molecules31050755

**Published:** 2026-02-24

**Authors:** Diana Stehlíková, Ágnes Farkas, Rita Filep, Nóra Papp, Dragica Purger, Jaroslav Tóth, Szilvia Czigle

**Affiliations:** 1 Department of Pharmacognosy and Botany, Faculty of Pharmacy, Comenius University Bratislava, Odbojárov 10, 832-32 Bratislava, Slovakia; stehlikova25@uniba.sk; 2 Department of Pharmacognosy, Faculty of Pharmacy, University of Pécs, Rókus u. 2, 7624 Pécs, Hungary

**Keywords:** unifloral honey, *Robinia pseudoacacia* L., antioxidant activity, pollen analysis, proline content, physicochemical properties, Pfund scale, total polyphenols, flavonoids, phenolic acids

## Abstract

The pseudoacacia species (*Robinia pseudoacacia* L.) are flowering plants that produce nectar and can be the sources of unifloral honey. *Robinia* is native to North America and is invasive in several European countries. The aim of this work was to determine and compare the physicochemical parameters, proline content, and antioxidant capacity of pseudoacacia honey collected from different locations in two central European countries (Hungary and Slovakia). The botanical origin of each honey sample was verified by melissopalynological analysis. The color intensity was determined using the Pfund scale. The antioxidant activity was determined with different spectrophotometric methods (DPPH, ABTS, and FRAP). The content of the total polyphenols, flavonoids, phenolic acids, and proline was quantified using spectrophotometric methods. The electrical conductivity, refractive index, and optical rotation were analyzed according to *European Pharmacopoeia, 12th edition*. Our study shows that the antioxidant capacity, and proline and phenolic content of unifloral pseudoacacia honeys can vary according to the geographical origin, polyphenol content, and the pollen profile in honey samples.

## 1. Introduction

The black locust (*Robinia pseudoacacia* L.), also known in Europe as pseudoacacia (false acacia), is a tree species classified as an invasive alien species in Europe. It originates from North America and was first introduced to Europe at the beginning of the 17th century as an ornamental tree planted in parks and along streets [1,2]. It was also planted to strengthen the soil to build fortresses [1], to obtain decay-resistant wood suitable for building boats or for construction, and as a melliferous plant that provides a valuable source of nectar for bees [3]. Acacia/pseudoacacia honey is classified as high-quality honey typical of Europe and the eastern United States [2].

In Slovakia, the black locust is not officially classified as invasive, but as an alien species with invasive potential, most widespread in the southern and western regions of the country. It does not require specific climatic or soil conditions for growth; it can easily adapt to dry climate and thrives on poor degraded soils, such as destroyed forests, polluted suburbs, or uncultivated fields. The tree is capable of rapid regeneration, especially after damage [1,3].

The black locust belongs to the family Fabaceae, which is characterized by the symbiotic relationship between its roots and nitrogen-fixing soil bacteria (rhizobia) that convert atmospheric nitrogen into the soil. However, this modifies soil quality by increasing the level of soil nitrogen and organic carbon, and by altering the soil’s pH. It negatively affects biodiversity [4], as nitrophilous and ruderal plant species begin to grow in such habitats. For this reason, the black locust was classified as an invasive plant species in Europe approximately 400 years after its introduction [1].

The black locust is characterized by the presence of bioactive compounds typical of the species and it is also rich in polyphenols. In the wood, flavonoids such as robinetin and (+)-dihydrorobinetin were identified in the highest concentrations. Stilbenes, including piceatannol and trace amounts of resveratrol, were also detected in the wood. Lectins have been identified in seeds, bark, roots, and leaves. Their biological function is to store proteins in the plant, although they are toxic. The seeds also contain catechin, epicatechin, and rutin [5].

There is a difference between the composition of flavonoids in its leaves and flowers. Flavonol 3,7-di-*O*-glycosides are the most abundant in flowers, mainly represented by kaempferol glycosides such as the toxic and thermolabile robinin, while flavone 7-*O*-glycosides, including acacetin, luteolin and apigenin glycosides, are predominant in leaves [2,6]. Some sesquiterpenes—(*Z*)-*β*-farnesene and (*E*)-*α*-bergamotene—as well as monoterpenes, including linalool, *δ*-3-carene, geraniol, terpinene-4-ol, (*E*)-*β*-ocimene, have been identified in flower extracts. However, the composition of the essential oil can vary depending on the geographical origin of the plant. Moreover, diterpenes, triterpenes, and unsaturated fatty acids have also been reported. Flowers are an important source of vitamin C, containing up to 40 mg of vitamin C per 100 g of flowers [5,6,7]. The distinctive feature of pseudoacacia honey is the presence of flavonol rhamnosides, originating from the nectar of black locust flowers [2].

As mentioned above, the black locust is a toxic plant and only flowers/petals are edible [7]. Compounds responsible for its toxicity include the alcaloid robinine, the glycoside robitin, and lectins (also known as toxoalbumins and glycoproteins) such as robin, ricin, and phazin, which are toxic to humans and livestock. Lectins cause the agglutination of erythrocytes, and their toxic effect occurs when they bind to carbohydrate moieties on the intestinal walls of mammals and cause nonspecific interference with nutrient absorption. Horses are particularly susceptible to lectin poisoning. Within one hour of ingestion, the livestock begins to exhibit symptoms such as anorexia, general weakness, depression, loss of appetite, paralysis of hindlimbs, abdominal pain, abnormal pulse, and bloody diarrhea [8].

In Slovakia, the Slovak Pharmaceutical Codex (2007) contained a monograph of black locust flower (Robiniae flos) as an herbal drug. The drug was defined as the dried flowers of *Robinia pseudoacacia* L., almost tasteless and sweet, with a honey-like aroma. The indication of the drug was to increase diuresis and the use of an infusion prepared from 2 g of the drug was recommended [9]. However, the Slovak Pharmaceutical Codex (2007) is no longer valid in Slovakia. Currently, the valid regulations include the European Pharmacopoeia and the European Medicines Agency, neither of which contains a monograph for Robiniae flos.

As the only edible part of the plant, flowers have traditionally been used in folk medicine to alleviate inflammation of the urinary and biliary tracts due to their diuretic, cholagogic, and spasmolytic properties [2]. Other traditional uses included the relief of cold symptoms, such as cough and fever, as well as relief from gastric spasms, rheumatic pain, migraines, and certain skin disorders. Laxative effects and cholesterol-lowering activity have also been reported. The phenolic compounds present in Robiniae flos exhibit antioxidant and antimicrobial properties [6], while the *O*-methylated flavone acacetin has been studied for its anticancer, antimalarial, antiangiogenic, and anti-inflammatory effects [10].

In Slovakia, black locust is considered the most important melliferous plant. It blooms in May and June [11,12] with characteristic white inflorescences and an intense sweet, pleasant fragrance and a sweet taste [6,7]. The optimal conditions for nectar production include cloudy weather and temperatures between 20 and 26 °C. Bees typically collect nectar toward the end of the flowering period, when the corolla, formed of five petals, can be opened with less effort, allowing easier access to nectar. The black locust is sensitive to spring frosts and heavy rains, which may destroy flowers [11,12].

The black locust provides nectar for bees that results in the production of pseudoacacia honey (also known as acacia honey or black locust honey), one of the most valuable honey types on the European market due to its highly appreciated sensory and physicochemical properties. One of the most important characteristics of pseudoacacia honey is that it remains in a liquid state for up to 3–4 years, showing the slowest crystallization among honey types due to its high fructose content and the high fructose-to-glucose ratio (average F/G = 1.61) [13,14]. Other typical properties include its very light color, weak floral odor similar to *Robinia* flowers [13] (especially in fresh honey [15]), and its mild, sweet, floral taste with low acidity similar to fresh fruit. Pseudoacacia honey is characterized by low enzymatic activity, electrical conductivity, acidity, and proline content but, on the other hand, by high levels of fructose and sucrose [13]. It contains only small amounts of antioxidant polyphenols, in contrast to darker honeys such as buckwheat (*Fagopyrum esculentum* Moench) or heather (*Calluna vulgaris* (L.) Hull) honey [5]. The content of *Robinia* pollen grains in pseudoacacia honey is low, and therefore it is classified as honey with under-represented pollen [16,17].

The specific polyphenolic profile of pseudoacacia honey cannot be precisely established, as it varies according to the geographical origin of the honey and the extraction method used. The most abundant polyphenols include 4-hydroxybenzoic acid, caffeic acid, *p*-coumaric acid, ferulic acid, chrysin, syringic acid, and apigenin. These polyphenols originate from nectar, propolis, and pollen of black locust flowers [17,18]. Other bioactive compounds in pseudoacacia honey include vitamins (A, C, and E), and some amino acids such as arginine, cysteine, aspartic and glutamic acid, and proline [18].

The aim of this work was to determine and compare the physicochemical parameters, proline content, and antioxidant capacity of pseudoacacia honey collected from different locations in two central European countries (Hungary and Slovakia). Antioxidant activity was determined using three different spectrophotometric methods: 1,1-diphenyl-2-picrylhydrazyl (DPPH), 2,2-azinobis-(3-ethylbenzothiazoline-6-sulfonate) (ABTS), also known as Trolox equivalent antioxidant capacity (TEAC), and ferric reducing antioxidant power (FRAP). We were also interested in the congruence and discriminating power of the applied spectrophotometric methods, as well as a comparison with the content of phenolic compounds (total polyphenols, flavonoids, and phenolic acids).

## 2. Results

Generally, a minimum of 45% of the characteristic pollen type is required to classify a honey as unifloral, unless otherwise specified, as in the case of pseudoacacia honey, for which at least 20% of the *Robinia pseudoacacia* pollen grains are required to establish uniflorality [19]. The pollen analysis showed that in this study, 11 out of the 14 honey samples were dominated by *Robinia pseudoacacia* pollen (more than 20%) and could, therefore, be treated as unifloral pseudoacacia honeys. There were two exceptions amongst Hungarian samples (A05 and A10) and one Slovak sample (A11), in which *Robinia* pollen was presented as secondary and important minor pollen. The samples A05 and A10 were dominated by *Brassica* pollen because the plant blooms simultaneously with *Robinia*. Sample A11 was dominated by pollen from the Lamiaceae family (Table 1).

Comparison of our samples with the intensity of the color of the Pfund scale showed that pseudoacacia honeys from Hungary and Slovakia had a water white color (Table 2).

The combination of non-enzymatic antioxidant assays provides the most reliable results; therefore, three different methods of total antioxidant capacity (TAC) were used to determine the antioxidant behavior of the pseudoacacia honey samples —DPPH, ABTS, and FRAP.

For the DPPH and ABTS assays, the results were expressed as SC_50_—the concentration of the sample extract that provides 50% inhibition of a free radical. The lower the SC_50_ value, the higher the antioxidant activity. The results were compared with ascorbic acid and Trolox solutions. For the FRAP assay, the results were expressed as the analogical amount of ascorbic acid (AA) at the initial sample concentration of 150 mg/mL, as well as compared with hyperoside (at the initial sample concentration of 20 mg/mL). The higher the AA value, the higher the antioxidant activity. The SC_50_ and AA values are summarized in Table 3.

The antioxidant activities are SC_50_ = 954–1872 mg/mL (DPPH), SC_50_ = 1101–1987 mg/mL (ABTS), and AA = 2.12–7.50 µmol/L (FRAP) (Table 3).

The content of some biologically active compounds was quantified by spectrophotometric methods—the total polyphenols with the Folin–Ciocalteu reagent, flavonoids with the AlCl_3_ reagent and phenolic acids with the Arnow reagent.

The content of total polyphenols (expressed as gallic acid equivalent) in the honey samples ranged from 1.11 ± 0.11 to 2.61 ± 0.18% (Table 4).

The content of flavonoids (expressed as hyperoside equivalent) varied from 0.27 ± 0.04 to 2.21 ± 0.15% (Table 4).

Similarly to the content of polyphenols and flavonoids, the content of phenolic acids (expressed as caffeic acid equivalent) was significantly different within the countries 0.23 ± 0.01 to 1.76 ± 0.10% (Table 4).

Some of the physicochemical parameters of honey were analyzed according to *Ph. Eur. 12* [22], which defines the requirements for the values obtained. The minimum limit for the refractive index is 1.487 (equivalent to a maximum water content of 20%), the maximum limit for electrical conductivity is 800 μS/cm, and the maximum limit for optical rotation is +0.6°.

The refractive index ranged from 1.487 ± 0.002 to 1.499 ± 0.000. The water content is closely related to the refractive index, and it ranged from 15.0 to 19.8%. Two out of the ten Hungarian samples were significantly different. (Table 5).

The optical rotation of the honey samples ranged from −2.9 ± 0.0 to 1.6 ± 0.0° (Table 5).

The electrical conductivity of pseudoacacia honey ranged from 125 ± 0.0 to 295 ± 0.0 μS/cm. The two previous parameters were significantly different within both countries. (Table 5).

All honey samples complied with *Ph. Eur. 12* requirements.

The proline content was determined using the spectrophotometric method established by the International Honey Commission, in which ninhydrin was used as a reagent [23]. The content of proline in honey serves as a criterion for assessing its quality, ripeness, authenticity and also its freshness [24]. For honey to be considered of good quality and unadulterated, the content of proline must exceed the limit of 180 mg of proline per kilogram of honey [23].

The proline content ranged from 191.0 ± 19.0 to 513.3 ± 8.8 mg/kg (Table 6). All samples complied with the internationally recognized requirement.

A low correlation was observed between the color intensity and antioxidant assays, and between the phenolic content and the DPPH assay. No significant correlation was observed between other parameters (Table 7). A medium correlation was found between proline content and the DPPH assay. Lower correlations were found between other parameters (Table 8).

## 3. Discussion

This study involved a total of 14 pseudoacacia honey samples obtained directly from beekeepers from various regions of Hungary and Slovakia. In both countries, black locust (*Robinia pseudoacacia* L.) is a widely distributed alien species that forms rich vegetations capable of rapid regeneration. [1,3].

The pollen analysis showed that 11 out of the 14 samples were unifloral and dominated by *Robinia* pollen. *Robinia* pollen grains are medium sized (26–50 μm), isopolar, have a circular outline, occur as monads, and do not aggregate. Their shape is spheroidal with a tricolporate aperture, a psilate and perforate exine [25]. Melissopalynology is a time-consuming method used to identify pollen grains in honey [16] and determine its botanical origin. However, it has several limitations, and a reliable classification requires its combination with organoleptic and physicochemical analyses [26]. Generally, honey is considered unifloral when the dominant pollen reaches at least 45% [20]. However, certain plant species produce under- or over-represented pollen [17]. Pseudoacacia honey belongs to the honey type with an under-represented pollen category [13,14]; therefore, an exception is applied. The national regulations in Croatia and Serbia established the unifloral threshold at 20%, and since there is no international standard, this study adopted the criterion of 20% [26].

Previous studies show that the pollen content of pseudoacacia honey varies widely. In the comprehensive study by Oddo et al. [13], which analyzed 715 European pseudoacacia honey samples, *Robinia* pollen content ranged from 7.0% to 59.5% (mean: 28.1%). Other studies reported values of 11.1–16.4% in Poland [27], 2–83% in Hungary [17], 45.27% on average in another Hungarian dataset [16], 5–57.9% in Spain [28], 11–70% in Croatia [29], and 54–60% in India [30].

In recent years, several alternative methods have been proposed for determining botanical and geographical origin, including near-infrared spectroscopy, mineral and amino acid profiling, electronic tongue and electronic nose to distinguish honey types according to the content of volatile compounds, and nuclear magnetic resonance (NMR) to distinguish nectar from honeydew honey [18,31]. GS-MS is also used to identify the taxonomic origin of honey based on the content of the volatile and semi-volatile compounds characteristic of different species of plants [32,33].

Physicochemical parameters defined by the European Pharmacopoeia, such as electrical conductivity, the refractive index, and specific rotation, are used to characterize honey [22]. Electrical conductivity and specific rotation are useful to distinguish blossom from honeydew honeys. The refractive index is correlated with the water content in honey, a critical quality parameter that affects fermentation stability [34]. Electrical conductivity also depends on the content of ash, acid components, proteins, and minerals [23,35]. The pharmacopoeial limit is a maximum of 800 μS/cm [22], and all samples in this study fell below this threshold. As expected, pseudoacacia honey exhibited low conductivity, consistent with values in other studies. Oddo et al. [13] found a mean of 0.16 ± 0.04 mS/cm in European samples. Similar values were reported for samples from Serbia, Slovenia, Bosnia and Herzegovina, Hungary, Romania and Croatia (0.13–0.172 mS/cm) [14,29,34,35,36,37,38].

Moisture content influences viscosity, crystallization, color, solubility [28] and maturity [34]. In general, the Codex Alimentarius [39] and the European Directive [40] set the limits for the moisture content to a maximum of 20%. All samples met this requirement. The moisture values reported for pseudoacacia honey in several countries are comparable to those found in this study, e.g., 17.1 ± 1.3% in the large dataset analyzed by Oddo et al. [13], and 15 to 18% in samples from Serbia, Bosnia and Herzegovina, Spain, Romania, Hungary, India, Poland, and Croatia [14,27,28,29,30,34,37,38].

The proline content is widely used as an indicator of honey ripeness, maturity, and potential sugar adulteration. It is one of the most abundant amino acids in honey [23,24,36]. Since proline determination is not legally regulated in most countries, the widely accepted reference value issued by the International Honey Commission (minimum 180 mg/kg) was adopted [24,41]. All samples met this requirement. Pseudoacacia honey is characterized by one of the lowest levels of proline compared to the highest levels in honeydew, heather, or chestnut honey [24,42]. Oddo et al. [13] reported a mean of 222 ± 58 mg/kg in 715 European samples. Studies from Hungary, Serbia, France, Estonia, and Poland have reported values of 252 ± 38 [42], 165.67 ± 119.89, 273.0 ± 65.9, 223.0 ± 83.9 [43], and 225.74 ± 43.88 mg/kg [44], respectively. The samples analyzed in this study exhibited a similar or even higher proline content.

The proline in honey originates both from plant pollen and the secretion of the pharyngeal glands of honeybees [24]. Consequently, its concentration can vary considerably within the same honey type depending on pollen composition, maturity, quality, and geographical origin. For this reason, proline content alone is not sufficiently reliable for botanical origin determination and should be supported by additional methods [44]. Several studies (Da Costa et al. [45] and Meda et al. [46]) reported correlations between the contents of proline and phenolic compounds, which contribute significantly to antioxidant activity. Some amino acids also exhibit antioxidant properties, strengthening this relationship. Antioxidant activity is closely related to the color of honey, as darker honeys usually contain higher amounts of polyphenols [47]. All samples in this study had Pfund values below 9, classifying them as “water white”, the lightest color category. A moderate correlation was observed between the proline content and the DPPH assay, while the correlation between proline and other antioxidant assays as well as the color intensity was low. No significant correlation was found with the phenolic content of honey, although the highest correlation was observed within the phenolic content for phenolic acids.

As noted above, honeys possess antioxidant activity. Pseudoacacia honey demonstrates lower antioxidant activity due to its relatively low levels of polyphenols. Antioxidant activity depends on botanical origin, climatic conditions, and polyphenolic profile [48,49]. In this study, antioxidant activity was assessed using three standard in vitro assays: DPPH, ABTS, and FRAP. Aqueous solutions (30%) were used to determine this activity by DPPH and ABTS, and were expressed as a SC_50_ value (mg/mL), while the FRAP results were expressed as ascorbic acid equivalents (μg/mL). For SC_50_ values, lower numbers indicate a higher antioxidant activity. In the literature sources, the total phenolic content in pseudoacacia honey is commonly expressed as milligrams of gallic acid (GA) per kg, i.e., in ppm (*m*/*m*) units. Compared with the samples analyzed in this study, foreign samples showed lower phenolic contents. For example, Slovenian, Croatian, Italian, Indian, Polish and German pseudoacacia honeys analyzed with the Folin–Ciocalteu reagent exhibited values ranging from 22.68 ± 1.98 to 627.56 ± 44.03 mg_GA_/kg [27,30,38,48,49,50]. The variation between studies can be attributed to differences in the analytical methods, as there is no standardized procedure for the determination of antioxidant capacity and each assay detects different groups of antioxidants [47]. Hungarian samples showed a DPPH IC_50_ of 61.76 ± 2.85 mg/mL [16]. Slovenian, Croatian, and Italian pseudoacacia honeys had DPPH IC_50_ values of 53.8 [50], 125.48 ± 42.04 [38], and 45.44 ± 0.04 mg/mL [49], respectively, and FRAP values of 71.0 [50], 36.1 ± 18.6 [38], and 79.5 ± 3.7 µM Fe(II) [49], respectively. Pseudoacacia honey originating from India showed an antioxidant activity of 52.27 ± 1.42%, expressed as the % of inhibition determined by the DPPH assay [30]. Polish samples showed 00.3 ± 0.0 mmol of TEAC/kg (expressed as the Trolox equivalent antioxidant capacity per kilogram of honey) (DPPH) and 0.6 ± 0.1 mmol Fe^2+^/kg (FRAP) [27]. In the study by Hassan et al. [48], DPPH and ABTS were expressed as SC_50_ with values of 13.62 ± 0.054 mg/mL (DPPH) and 44.37 ± 0.790 mg/mL (ABTS), while the FRAP value (measured at λ = 700 nm) was 1.366 ± 0.06 ABS_700_. The samples analyzed in this study showed different values of antioxidant activities from those reported in the literature. As noted above, antioxidant activity is strongly influenced by geographical origin, and honey from different regions may contain pollen from various plant species, thus affecting their phenolic and antioxidant profiles [47]. A low correlation was observed between color intensity and antioxidant assays. No significant correlation was found with the phenolic content of honey and antioxidant methods, although the highest correlation was observed within the phenolic content for the DPPH assay.

## 4. Materials and Methods

### 4.1. Honey Samples

**Figure 1:**
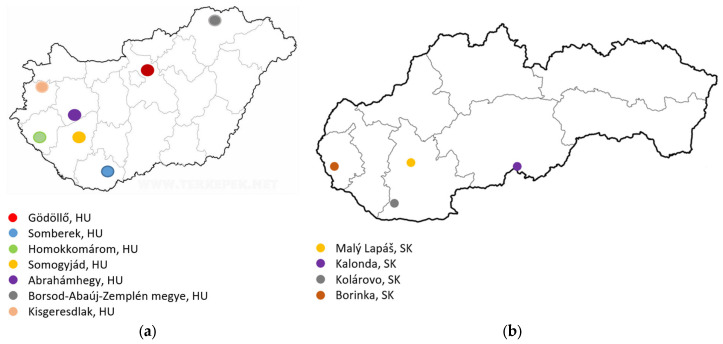
Places of origin of Hungarian (**a**), and Slovak (**b**) pseudoacacia honey.

**Figure 2:**
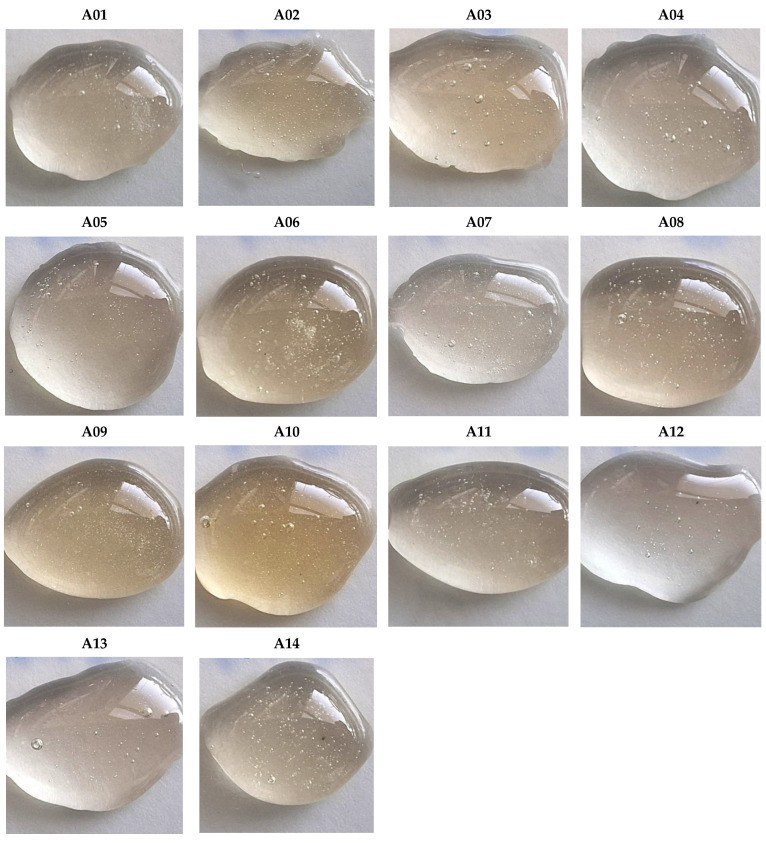
Hungarian- (A01–A10), and Slovak-origin (A11–A14) pseudoacacia honey samples: drops of fresh honey illustrating their very light color. The color intensity is calculated based on the absorbance of water solutions and expressed based on the Pfund scale, which classified all samples as “water white” (Table 2) [21].

Pseudoacacia honeys were collected from various locations in both Hungary and Slovakia. Hungarian-origin pseudoacacia honeys were collected from Homokkomárom (A01), Ábrahámhegy (A02), Borsod-Abaúj-Zemplén megye (A03), Somogyjád (A04), Kisgeresdlak (A05), Gödöllő (A06), Ábrahámhely (A07), Somberek (A08), Somogyjád (A09), and Kisgeresdlak (A10). Slovak-origin pseudoacacia honeys were collected from Malý Lapáš (A11), Kalonda (A12), Kolárovo (A13), and Borinka (A14) (Figure 1 and Figure 2). The samples were harvested in May–June of 2018–2021 and stored at room temperature (21 °C) in the dark until analysis.

### 4.2. Melissopalynological Analysis

The honey samples were purchased in 2018–2021, directly from beekeepers, who identified the samples as pseudoacacia honey. The botanical origin of each honey sample was checked with microscopic pollen analysis. Honey samples, when fluid, were stirred thoroughly. In case they contained large crystals, they were heated in a 40 °C water bath, until fluid, then stirred. A total of 10 g of honey was measured into centrifuge tubes (50 mL volume); 20 mL of distilled water was added, and then it was vortexed with Combi-spin FVL-2400N (Biocenter Ltd., Szeged, Hungary). The solution was centrifuged at 3000 rpm for 10 min with a Neofuge 15R centrifuge (Lab-Ex Ltd., Budapest, Hungary). The supernatant was decanted, and then 10 mL of distilled water was added to the sediment; this mixture was centrifuged again at 3000 rpm for 10 min and decanted. Any remaining fluid was removed by setting the centrifuge tubes on filter paper. A frame, of the size of the cover glass, was drawn on each microscope slide with a paint marker (Edding 750, D. Ledermann & Co. GmbH, Bautzen, Germany); then, the microscope slides were placed on a heating plate (OTS 40, Tiba Ltd., Győr, Hungary) set at 40 °C. A total of 0.25 mL of distilled water was added to the sediment in the centrifuge tube, then vortexed. A volume of 20 μL of the pollen suspension was pipetted onto the microscope slide within the frame. The water was allowed to evaporate from the slide on the heating plate. The pollen preparation was mounted in fuchsine glycerol jelly (fuchsine was added to Kaiser’s glycerol jelly). Pollen preparations were studied with a Nikon Eclipse E200 microscope equipped with a Michrome 20 MP CMOS digital camera (Auro-Science Consulting Ltd., Budapest, Hungary), and microphotos were taken with the Capture 1.2 software at 400× magnification. At least 500 pollen grains per honey sample were counted and the source plants were identified at the species or at least at the family level. Identification was performed using a pollen atlas (*Beekeeping Pollen Atlas, 1.0*, 2017). The relative frequency for each type of pollen was calculated as a percentage of the total number of pollen grains [13].

### 4.3. Color Intensity

The color intensity was determined according to Ferreira et al. [51]. Pseudoacacia honey samples were diluted to 50% (*m*/*v*) with distilled water, mixed and centrifuged at 3200 rpm/5 min (centrifuge Hettich Universal 320 R, Hettich GmbH & Co.KG, Tuttlingen, Germany). The absorbance was measured at 635 nm using a SPECTRO-V/UV 1/2 spectrophotometer (MRC, Holon, Israel), and the color intensity was determined using the Pfund scale [21], using the following equation:

Pfund scale=−38.70+371.39×A

A = absorbance.

### 4.4. DPPH Method

The DPPH assay was performed according to the method reported by Vundać et al. [52]. A total of 1.8 mL of DPPH methanol solution was added to 0.2 mL of various concentrations of honey water dilutions, prepared from 30% water solutions of the honey samples. The solution was then thoroughly shaken and left to react in the dark at room temperature. The absorbance of the solution was measured after 30 min. Methanol (1.8 mL) and honey extracts (0.2 mL) were used as a blank; DPPH solution (1.8 mL) and methanol (0.2 mL) were used as the negative control. The positive control was 1.8 mL of DPPH solution and 0.2 mL of ascorbic acid/Trolox solution. The antioxidant activity (%) was calculated using the absorption values of the samples versus negative control at 517 nm (Genesys^TM^10 spectrophotometer, Thermo Electron Corporation, Cambridge, UK), and the results were expressed as SC_50_ (concentration of the sample dilution providing 50% inhibition of the DPPH radical) using linear regression for samples with a lower antioxidant capacity. The assay was carried out in triplicate (replicate sampling of each of the 14 honey samples) [53].

### 4.5. ABTS Method

The ABTS assay was performed according to the method reported by Re et al. [54]. A total of 2 mL of ABTS radical solution was added to 0.1 mL of various concentrations of honey water dilutions, prepared from 30% water solutions of the honey samples. The solution was then thoroughly shaken and left to react in the dark at room temperature. The absorbance of the solution was measured after 5 min. Ethanol (2 mL) and honey extracts (0.1 mL) were used as a blank; ABTS solution (2 mL) and methanol (0.1 mL) were used as the negative control. The positive control was 2 mL of ABTS solution and 0.1 mL of ascorbic acid/Trolox solution. The antioxidant activity (%) was calculated using the absorption values of the samples versus the negative control at 734 nm (Genesys^TM^10 spectrophotometer, Thermo Electron Corporation, Cambridge, UK), and the results were expressed as SC_50_ (concentration of the sample dilution providing 50% inhibition of the ABTS radical) using linear regression for samples with a lower antioxidant capacity. The assay was carried out in triplicate (replicate sampling of each of the 14 honey samples) [53].

### 4.6. FRAP Method

The FRAP assay was performed according to the method reported by Benzie and Strain [55]. A total of 3 mL of the FRAP reagent was added to 0.1 mL of various concentrations of honey water dilutions, prepared from 30% water solutions of the honey samples. The solution was then thoroughly shaken and left to react in the dark at room temperature. The absorbance of the solution was measured after 5 min. The FRAP reagent was used as a blank. Hyperoside was used as a positive control. The results were expressed as an analogical amount of ascorbic acid (μg/mL) and calculated using the absorption values of the samples at 593 nm (Genesys^TM^10 spectrophotometer, Thermo Electron Corporation, Cambridge, UK). The assay was carried out in triplicate (replicate sampling of each of the 14 honey samples) [53].

### 4.7. Quantification of Total Polyphenolic Compounds Expressed as Gallic Acid

The quantification of the total polyphenols was performed following the analytical procedure described by Singleton et al. [56]. This method is a modified spectrophotometric Folin–Ciocalteu method. Briefly, 5 g of honey was mixed with distilled water up to 10.0 mL. Then, 1.0 mL of the honey solution (50% *m*/*v*) was mixed with 1.0 mL of the Folin–Ciocalteu reagent. After 3 min, 1.0 mL of 10.0% sodium carbonate (*m*/*v*) solution was added to the mixture and adjusted to 10.0 mL with distilled water. The reaction was kept in the dark for 90 min, after which the absorbance was read at 725 nm using a Genesys^TM^10 spectrophotometer (Thermo Electron Corporation, Cambridge, UK). The results are reported as the mean ± standard deviation and expressed as the percentage of the total polyphenolic compounds expressed as gallic acid based on a calibration curve (k = 23.4, r^2^ = 0.9997). All the samples were analyzed in triplicate (replicate sampling of each of the 14 honey samples) [47].

### 4.8. Quantification of Flavonoids Expressed as Hyperoside

The quantification of flavonoids was performed following the modified spectrophotometric analytical procedure [57]. For the quantification of flavonoids, the honey samples were diluted to 50% (*m*/*v*) with distilled water. Firstly, 1.0 mL of the honey solution was mixed with 1.0 mL of a hexamethylenetetramine solution (5 g/L), 20.0 mL of acetone and 2.0 mL of hydrochloric acid, and the mixture was boiled for 30 min. After liquid–liquid extraction with ethyl acetate, 1.0 mL of the 10% (*m*/*v*) aluminum chloride reagent was added to the combined ethyl acetate extract and diluted to 25.0 mL with a 5% (*v*/*v*) solution of glacial acetic acid in methanol. After 30 min, the absorbance was measured at 425 nm using a Genesys^TM^10 spectrophotometer (Thermo Electron Corporation, Cambridge, UK). The results are reported as the mean ± standard deviation and expressed as the percentage of flavonoids expressed as hyperoside based on a calibration curve (k = 34.5, r^2^ = 0.998). All the samples were analyzed in triplicate (replicate sampling of each of the 14 honey samples) [47].

### 4.9. Quantification of Phenolic Acids Expressed as Caffeic Acid

For the quantification of phenolic acids expressed as caffeic acid, according to Arnow’s method [58], we used 50% ethanol to prepare diluted honey samples (50% *m*/*v*). First, 1.0 mL of the honey ethanolic solution was mixed with 2.0 mL of 0.5 M hydrochloric acid, 2.0 mL of Arnow’s reagent (containing sodium nitrite), and 2.0 mL of an 8.5% (*m*/*v*) sodium hydroxide solution. The volume was increased to 10.0 mL with distilled water. The mixture was shaken and the absorbance was read at 505 nm using a Genesys^TM^10 spectrophotometer (Thermo Electron Corporation, Cambridge, UK). The results are reported as the mean ± standard deviation and expressed as the percentage of phenolic acids expressed as caffeic acid based on a calibration curve (k = 44.5, r^2^ = 0.9998). All the samples were analyzed in triplicate (replicate sampling of each of the 14 honey samples) [47].

### 4.10. Refractive Index and Water Content

For the determination of the refractive index by *Ph. Eur. 12* [22], we used homogenized honey samples (digital refractometer, ORM 1RS, Kern & Sohn, Balingen, Germany). All the samples were analyzed in triplicate (replicate sampling of each of the 14 honey samples). The water content was determined according to the table provided in the monograph of *Ph. Eur. 12* [22] and expressed as a percentage (*m*/*m*).

### 4.11. Electrical Conductivity

For the determination of electrical conductivity by *Ph. Eur. 12* [22], we used 20% (*m*/*v*) water solutions of honey samples (conductivity meter, OK 102/1, Radelkis, Hungary). All the samples were analyzed in triplicate (replicate sampling of each of the 14 honey samples).

### 4.12. Polarimetry

For the determination of the optical rotation by *Ph. Eur. 12* [22], we used 20% (*m*/*v*) water solutions of honey samples (polarimeter, Zuzi 404-LED, Auxilab, Beriáin, Navarra, Spain). All the samples were analyzed in triplicate (replicate sampling of each of the 14 honey samples).

### 4.13. Quantification of Proline

The quantification of proline was carried out according to the procedure of the International Honey Commission [23]. For the quantification, we used 5% (*m*/*v*) water solutions of honey samples. In total, 0.5 mL of the sample solution, 0.5 mL of water (blank test) and 0.5 mL of proline standard solution were diluted with formic acid (1.0 mL), and ninhydrin solution (1.0 mL) and shaken vigorously for 15 min. For another 15 min, the samples were placed in a boiling water bath and then transferred to a hot water bath (70 °C) for 10 more minutes. An isopropyl alcohol water solution (50% *v*/*v*, 5.0 mL) was added, and the absorbance was measured using a SPECTRO-V/UV 1/2 spectrophotometer (MRC, Holon Israel) 45 min after removing from the hot water bath at 511 nm. All the samples were analyzed in triplicate (replicate sampling of each of the 14 honey samples) and the proline content was expressed as mg of proline per kg of honey [23].

### 4.14. Statistical Analysis

All measurements were performed in three replicates of 14 pseudoacacia honey samples. The data were analyzed using Welch’s *t*-test. Differences were considered statistically significant at *p* ≤ 0.05. The Holm method was applied as the multiple testing correction method to counteract the problem of multiple comparisons. Table 3, Table 4, Table 5 and Table 6 show the data with this correction applied. Statistical analyses were carried out using GraphPad Prism, version 10.6.1.

Pearson’s correlation coefficient was measured to find the association between color intensity, total polyphenolic content, flavonoid content, phenolic acid content, antioxidant activities and proline content using Microsoft Excel.

## 5. Conclusions

This study examined fourteen pseudoacacia honey samples from Hungary and Slovakia in terms of their botanical, physicochemical, and antioxidant properties. The melissopalynological analysis confirmed that 11 out of 14 samples were unifloral. The physicochemical parameters, including the refractive index, moisture, electrical conductivity, and optical rotation, were consistent with the international quality standards defined by *Ph. Eur. 12*. The proline content was relatively low, as is typical for pseudoacacia honey, yet it also met the international standards. The antioxidant activity was low, as expected for light-colored pseudoacacia honey, and showed a moderate correlation with the proline content and color intensity, while correlations with phenolic contents were low. These findings reveal the influence of botanical and geographical origin, the representation of different pollen types, and honey maturity on the quality and bioactive properties of pseudoacacia honey, emphasizing the need to combine multiple analytical methods for a reliable assessment of its characteristics.

## Figures and Tables

**Table 1:** Pollen analysis of Hungarian- and Slovak-origin pseudoacacia honey.

			Pollen Type—Relative Frequency (%) ^a^					
Sample Code	Geographical Origin	Date of Collection	*Robinia*	*Brassica*	*Helianthus*	Lamiaceae	*Castanea*	Other
A01	Homokkomárom, HU	2018	23.3	40.1	0.7	-	3.8	32.1
A02	Ábrahámhegy, HU	2018	35.5	7.1	0.1	-	3.5	53.8
A03	B-A-Z megye, HU	2018	41.2	19.3	0.1	-	4.5	34.9
A04	Somogyjád, HU	2019	33.1	26.3	-	-	7.5	33.1
A05	Kisgeresdlak, HU	2019	19.3	40.3	0.4	-	8.8	31.2
A06	Gödöllő, HU	2019	23.7	34.3	0.4	-	5.7	35.9
A07	Ábrahámhely, HU	2020	34.8	16.3	-	-	7.3	41.6
A08	Somberek, HU	2020	22.6	53.0	0.2	-	5.4	18.8
A09	Somogyjád, HU	2021	44.1	6.7	0.7	-	3.4	45.1
A10	Kisgeresdlak, HU	2021	6.9	44.4	-	-	-	48.7
A11	Malý Lapáš, SK	2021	12.5	12.4	-	38.0	5.5	31.6
A12	Kalonda, SK	2021	30.0	22.6	-	-	4.5	42.9
A13	Kolárovo, SK	2021	31.0	14.0	15.2	-	7.2	32.6
A14	Borinka, SK	2021	20.2	30.3	-	-	6.6	42.9

Hungarian- (A01–A10) and Slovak-origin (A11–A14) pseudoacacia honey samples. ^a^ Evaluation of pollen samples: predominant pollen: >45% of the pollen grains counted; secondary pollen: 16–45%; important minor pollen 3–15%; and minor pollen < 3% [20].

**Table 2:** Color intensity of Hungarian- and Slovak-origin pseudoacacia honey.

Sample Code	Geographical Origin	Date of Collection	Absorbance ^a^ (A)	Color Intensity Pfund Scale (mm) ^a^	Color Name ^a^
A01	Homokkomárom, HU	2018	0.062	−15.7	water white
A02	Ábrahámhegy, HU	2018	0.124	7.4	water white
A03	B-A-Z megye, HU	2018	0.084	−7.5	water white
A04	Somogyjád, HU	2019	0.067	−13.8	water white
A05	Kisgeresdlak, HU	2019	0.051	−19.9	water white
A06	Gödöllő, HU	2019	0.042	−23.3	water white
A07	Ábrahámhely, HU	2020	0.060	−16.4	water white
A08	Somberek, HU	2020	0.104	−0.1	water white
A09	Somogyjád, HU	2021	0.078	−8.8	water white
A10	Kisgeresdlak, HU	2021	0.108	2.3	water white
A11	Malý Lapáš, SK	2021	0.090	−4.4	water white
A12	Kalonda, SK	2021	0.050	−19.2	water white
A13	Kolárovo, SK	2021	0.100	−0.7	water white
A14	Borinka, SK	2021	0.064	−14.0	water white

Hungarian- (A01–A10) and Slovak-origin (A11–A14) pseudoacacia honey samples. ^a^ Color intensity and color name calculated based on the absorbance (A) of 50% water solutions of honey samples (measured at λ = 635 nm) and expressed based on the Pfund scale (mm): water white < 9; extra white 9–17; white 18–34; extra-light amber 35–50; light amber 51–85; amber 86–114; and dark amber > 114 [21].

**Table 3:** Antioxidant activity of Hungarian- and Slovak-origin pseudoacacia honey.

Sample Code	Geographical Origin	Date of Collection	DPPH ^a^ SC_50_ (mg/mL)	ABTS ^b^ SC_50_ (mg/mL)	FRAP ^c,d^ (µg/mL)
A01	Homokkomárom, HU	2018	1428 ± 50 ^ns^	1312 ± 50 **	3.43 ± 0.01 *
A02	Ábrahámhegy, HU	2018	1110 ± 48 **	1336 ± 50 **	4.95 ± 0.02 ^ns^
A03	B-A-Z megye, HU	2018	1161 ± 46 *	1101 ± 43 ***	4.41 ± 0.02 ^ns^
A04	Somogyjád, HU	2019	1286 ± 48 ^ns^	1401 ± 52 ^ns^	2.12 ± 0.01 ***
A05	Kisgeresdlak, HU	2019	1379 ± 50 ^ns^	1470 ± 54 ^ns^	2.54 ± 0.01 ***
A06	Gödöllő, HU	2019	1033 ± 41 ***	1987 ± 57 ***	2.54 ± 0.01 ***
A07	Ábrahámhely, HU	2020	1287 ± 45 ^ns^	1916 ± 58 ***	5.81 ± 0.03 ***
A08	Somberek, HU	2020	1337 ± 50 ^ns^	1732 ± 57 *	4.02 ± 0.02 ^ns^
A09	Somogyjád, HU	2021	1377 ± 43 ^ns^	1825 ± 54 ***	7.50 ± 0.04 ***
A10	Kisgeresdlak, HU	2021	1872 ± 52 ***	1314 ± 50 **	5.51 ± 0.03 **
A11	Malý Lapáš, SK	2021	1130 ± 45 ^ns^	1250 ± 46 ^ns^	5.30 ± 0.03 ^ns^
A12	Kalonda, SK	2021	1430 ± 53 ^###^	1569 ± 52 ^###^	3.22 ± 0.02 ^##^
A13	Kolárovo, SK	2021	954 ± 39 ^##^	1112 ± 44 ^#^	3.72 ± 0.02 ^ns^
A14	Borinka, SK	2021	1110 ± 42 ^ns^	1207 ± 52 ^ns^	6.40 ± 0.04 ^###^
ascorbic acid			0.02 ± 0.00	0.02 ± 0.00	–
Trolox			0.02 ± 0.00	0.28 ± 0.00	–
hyperoside			–	18.44 ± 1.42	5.44 ± 0.42 ^e^

Hungarian- (A01–A10) and Slovak-origin (A11–A14) pseudoacacia honey samples. ^a^ DPPH—antiradical power; ^b^ ABTS—Trolox equivalent antioxidant capacity; and ^c^ FRAP—ferric reducing antioxidant power. Data are means ± standard deviations of three replicate samplings; data with superscripted symbols (*, **, and *** for ten Hungarian samples, and ^#^, ^##^, and ^###^ for four Slovak samples) mean significant differences among honeys within one country: *,^#^ *p* < 0.05, **,^##^ *p* < 0.01, and ***,^###^ *p* < 0.001; and the superscripted symbol (^ns^) means non-significant difference among honeys within one country; ^d^ ascorbic acid (AA) value at the initial sample concentration of 150 mg/mL; ^e^ AA value at the initial sample concentration of 20 mg/mL; and standards (ascorbic acid, Trolox, and hyperoside).

**Table 4:** Content of total polyphenols, flavonoids, and phenolic acids of Hungarian- and Slovak-origin pseudoacacia honey.

Sample Code	Geographical Origin	Date of Collection	Total Polyphenols as Gallic Acid (%)	Flavonoids as Hyperoside (%)	Phenolic Acids as Caffeic Acid (%)
A01	Homokkomárom, HU	2018	1.54 ± 0.12 ^ns^	1.07 ± 0.10 ^ns^	0.55 ± 0.04 ^ns^
A02	Ábrahámhegy, HU	2018	1.18 ± 0.10 **	0.88 ± 0.05 ^ns^	0.48 ± 0.04 ^ns^
A03	B-A-Z megye, HU	2018	1.45 ± 0.11 ^ns^	0.86 ± 0.08 ^ns^	0.57 ± 0.04 ^ns^
A04	Somogyjád, HU	2019	1.22 ± 0.11 *	0.27 ± 0.04 ***	0.46 ± 0.04 ^ns^
A05	Kisgeresdlak, HU	2019	1.35 ± 0.14 ^ns^	0.36 ± 0.04 ***	0.45 ± 0.04 ^ns^
A06	Gödöllő, HU	2019	2.51 ± 0.12 ***	1.63 ± 0.06 ***	1.39 ± 0.04 ***
A07	Ábrahámhely, HU	2020	1.23 ± 0.11 *	0.56 ± 0.04 **	0.36 ± 0.03 **
A08	Somberek, HU	2020	2.12 ± 0.12 **	1.53 ± 0.04 ***	0.23 ± 0.01 ***
A09	Somogyjád, HU	2021	2.18 ± 0.16 **	1.57 ± 0.04 ***	1.26 ± 0.04 ***
A10	Kisgeresdlak, HU	2021	1.11 ± 0.11 **	0.59 ± 0.06 **	0.54 ± 0.04 ^ns^
A11	Malý Lapáš, SK	2021	2.27 ± 0.17 ^ns^	1.66 ± 0.04 ^ns^	1.44 ± 0.10 ^ns^
A12	Kalonda, SK	2021	2.02 ± 0.12 ^ns^	0.89 ± 0.07 ^ns^	1.32 ± 0.10 ^ns^
A13	Kolárovo, SK	2021	2.61 ± 0.18 ^##^	2.21 ± 0.15 ^###^	1.76 ± 0.10 ^##^
A14	Borinka, SK	2021	1.12 ± 0.10 ^###^	0.57 ± 0.04 ^##^	0.52 ± 0.04 ^###^

Hungarian- (A01–A10) and Slovak-origin (A11–A14) pseudoacacia honey samples; data are means ± standard deviations of three replicate samplings; data with superscripted symbols (*, **, and *** for ten Hungarian samples, and ^##^, and ^###^ for four Slovak samples) mean significant differences among honeys within one country: * *p* < 0.05, **,^##^ *p* < 0.01, and ***,^###^ *p* < 0.001; and the superscripted symbol (^ns^) means non-significant difference among honeys within one country.

**Table 5:** Refractive index, water content, polarimetry, and electrical conductivity of Hungarian- and Slovak-origin pseudoacacia honey.

Sample Code	Geographical Origin	Date of Collection	Refractive Index	Water Content (%)	Polarimetry (α [°])	Electrical Conductivity (µS/cm)
A01	Homokkomárom, HU	2018	1.496 ± 0.002 ^ns^	16.2	−2.3 ± 0.0 ^ns^	125 ± 0.0 ***
A02	Ábrahámhegy, HU	2018	1.499 ± 0.000 ***	15.0	−2.0 ± 0.0 ***	295 ± 0.0 ***
A03	B-A-Z megye,HU	2018	1.490 ± 0.002 ^ns^	18.6	−2.6 ± 0.0 ***	225 ± 0.0 *
A04	Somogyjád,HU	2019	1.488 ± 0.003 ^ns^	19.4	−2.3 ± 0.0 ^ns^	160 ± 0.0 **
A05	Kisgeresdlak, HU	2019	1.494 ± 0.004 ^ns^	17.0	−2.7 ± 0.0 ***	165 ± 0.0 **
A06	Gödöllő, HU	2019	1.495 ± 0.000 ^ns^	16.6	−2.2 ± 0.0 ^ns^	160 ± 0.0 **
A07	Ábrahámhely, HU	2020	1.487 ± 0.002 ^ns^	19.8	−2.6 ± 0.0 ***	255 ± 0.0 ***
A08	Somberek, HU	2020	1.489 ± 0.006 ^ns^	19.0	−1.8 ± 0.0 ***	225 ± 0.0 *
A09	Somogyjád, HU	2021	1.490 ± 0.003 ^ns^	18.6	−2.2 ± 0.0 ^ns^	144 ± 0.0 ***
A10	Kisgeresdlak, HU	2021	1.495 ± 0.001 ^ns^	16.4	−2.1 ± 0.0 **	245 ± 0.0 ***
A11	Malý Lapáš, SK	2021	1.495 ± 0.001 ^ns^	16.8	−2.9 ± 0.0 ^##^	190 ± 0.0 ^ns^
A12	Kalonda, SK	2021	1.492 ± 0.004 ^ns^	17.8	−2.8 ± 0.0 ^#^	128 ± 0.0 ^###^
A13	Kolárovo, SK	2021	1.490 ± 0.000 ^ns^	18.6	−1.6 ± 0.0 ^##^	260 ± 0.0 ^#^
A14	Borinka, SK	2021	1.489 ± 0.006 ^ns^	19.0	−1.8 ± 0.0 ^#^	250 ± 0.0 ^#^

Hungarian- (A01–A10) and Slovak-origin (A11–A14) pseudoacacia honey samples; data are means ± standard deviations of three replicate samplings; data with superscripted symbols (*, **, and *** for ten Hungarian samples, and ^#^, ^##^, and ^###^ for four Slovak samples) mean significant differences among honeys within one country: *,^#^ *p* < 0.05, **,^##^ *p* < 0.01, and ***,^###^ *p* < 0.001; and the superscripted symbol (^ns^) means non-significant difference among honeys within one country.

**Table 6:** Proline content of Hungarian- and Slovak-origin pseudoacacia honey.

Sample Code	Geographical Origin	Date of Collection	Proline (mg/kg)
A01	Homokkomárom, HU	2018	191.0 ± 19.0 *
A02	Ábrahámhegy, HU	2018	382.0 ± 11.7 ^ns^
A03	B-A-Z megye, HU	2018	259.6 ± 14.6 ^ns^
A04	Somogyjád, HU	2019	258.1 ± 13.1 *
A05	Kisgeresdlak, HU	2019	326.6 ± 11.7 ^ns^
A06	Gödöllő, HU	2019	275.6 ± 24.8 ^ns^
A07	Ábrahámhely, HU	2020	438.9 ± 16.0 *
A08	Somberek, HU	2020	447.7 ± 7.3 ***
A09	Somogyjád, HU	2021	303.3 ± 11.7 ^ns^
A10	Kisgeresdlak, HU	2021	513.3 ± 8.8 ***
A11	Malý Lapáš, SK	2021	240.6 ± 10.2 ^ns^
A12	Kalonda, SK	2021	306.2 ± 14.6 ^ns^
A13	Kolárovo, SK	2021	250.8 ± 23.3 ^ns^
A14	Borinka, SK	2021	298.9 ± 4.4 ^ns^

Hungarian- (A01–A10) and Slovak-origin (A11–A14) pseudoacacia honey samples; data are means ± standard deviations of three replicate samplings; data with superscripted symbols (* and *** for ten Hungarian samples) mean significant differences among honeys within one country: * *p* < 0.05 and *** *p*< 0.001; and the superscripted symbol (^ns^) means non-significant difference among honeys within one country.

**Table 7:** Correlation between color intensity, total polyphenols, flavonoids, and phenolic acids content and antioxidant activities.

	Pearson Correlation Coefficient (r) with			
Antioxidant Methods	Color Intensity	Total Polyphenols as Gallic Acid (%)	Flavonoids as Hyperoside (%)	Phenolic Acids as Caffeic Acid (%)
DPPH	0.055	−0.404	−0.446	−0.379
ABTS	−0.423	0.254	0.075	−0.003
FRAP	0.377	−0.134	0.090	−0.012

**Table 8:** Correlation between color intensity, total polyphenols, flavonoids, and phenolic acids content and proline content.

	Pearson Correlation Coefficient (r) with						
Proline Content (mg/kg)	DPPH	ABTS	FRAP	Color Intensity	Total Polyphenols as Gallic Acid (%)	Flavonoids as Hyperoside (%)	Phenolic Acids as Caffeic Acid (%)
	−0.534	0.320	−0.316	0.393	−0.350	−0.280	−0.471

## Data Availability

The data presented in this study are available on request from the corresponding author.

## Abbreviations

The following abbreviations are used in this manuscript:

*Ph. Eur. 12*	*European Pharmacopoeia, 12th edition*
